# Specific In Vivo Staining of Astrocytes in the Whole Brain after Intravenous Injection of Sulforhodamine Dyes

**DOI:** 10.1371/journal.pone.0035169

**Published:** 2012-04-11

**Authors:** Florence Appaix, Sabine Girod, Sylvie Boisseau, Johannes Römer, Jean-Claude Vial, Mireille Albrieux, Mathieu Maurin, Antoine Depaulis, Isabelle Guillemain, Boudewijn van der Sanden

**Affiliations:** 1 Grenoble Institute of Neuroscience, Inserm U836, Grenoble, France; 2 Université Joseph Fourier, Grenoble, France; 3 Laboratoire Interdisciplinaire de Physique, CNRS UMR 5588, Saint Martin d'Hères, France; Centre national de la recherche scientifique, University of Bordeaux, France

## Abstract

Fluorescent staining of astrocytes without damaging or interfering with normal brain functions is essential for intravital microscopy studies. Current methods involved either transgenic mice or local intracerebral injection of sulforhodamine 101. Transgenic rat models rarely exist, and in mice, a backcross with GFAP transgenic mice may be difficult. Local injections of fluorescent dyes are invasive. Here, we propose a non-invasive, specific and ubiquitous method to stain astrocytes *in vivo*. This method is based on iv injection of sulforhodamine dyes and is applicable on rats and mice from postnatal age to adulthood. The astrocytes staining obtained after iv injection was maintained for nearly half a day and showed no adverse reaction on astrocytic calcium signals or electroencephalographic recordings *in vivo*. The high contrast of the staining facilitates the image processing and allows to quantify 3D morphological parameters of the astrocytes and to characterize their network. Our method may become a reference for *in vivo* staining of the whole astrocytes population in animal models of neurological disorders.

## Introduction

*In vivo* studies of astrocytes have gained importance over the last ten years. These cells have been found to be implicated in neurotransmission with the concept of the ‘tripartite synapse’ [1], in neurovascular coupling [2], [3], [4] and in angiogenesis [5]. Possible dysfunction of astrocytes has been suggested to be critical in several neurological diseases [6], [7], [8].

Two-photon laser scanning microscopy (TPLSM) *in vivo* allows imaging of biological tissue with micrometric spatial resolution and gives access to numerous observations at the cellular level [9]. TPLSM has made possible *in vivo* studies of astrocytes in the intact brain [10], [11], [12] and has opened a new field of dynamic and functional studies on neuron-astrocytes and astrocytes-vasculature interactions [13]. To this aim, transgenic mice expressing fluorescent protein under astrocyte specific promoter (human glial fibrillary acidic protein, S100…) have been developed [14]. However, crossing mice carrying two transgenes, using e.g. the cre lox system [15], with GFAP transgenic mice is not trivial. Further, rats are often used to study normal brain functions or neuropathologies, but transgenic rat models are difficult to generate [16], [17]. Thus, alternative methods to stain astrocytes *in vivo* are necessary. In 2004, a method was proposed for *in vivo* astrocytes staining in the neocortex of anesthetized rats and mice using sulforhodamine 101 (SR101) [12]. The dye was directly applied on the brain cortex for local astrocytes staining via the gap junctions or intracerebrally injected for deeper labeling. However, this technique requires invasive introduction of a patch pipette in the brain tissue which may interfere with physiological functions.

Recently, we found that sulforhodamine B (SRB) injected intravenously (iv) specifically stained astrocytes in mice neocortex [18]. SRB is a non-toxic molecule with an oral LD_50_ = 10300 mg/kg of body weight [19], [20]. Moreover, sulforhodamine dyes family and in particular SR101, SRB and SRG show a high two-photon absorption cross section (σ_TPE_∼150 GM) [21] with an emission and a two-photon absorption wavelength located in the spectral region of interest for deep bio-imaging: 680–1040 nm. The possibility to use these fluorescent dyes in a non-invasive way makes them a powerful tool for *in vivo* studies of astrocytes.

The aim of the present study was to characterize the astrocytes staining obtained after iv injection of sulforhodamine dyes (SR101, SRB or SRG). We performed TPLSM on mice and rats from newborn stages to adulthood in acute brain slices and *in vivo*. We verified the compatibility of this method with astrocytes calcium imaging and electroencephalography (EEG) recordings in freely moving rats. Finally, a three dimensional (3D) analysis of the astrocytic network has been done to quantify astrocytes distribution and density in the neocortex. The use of this 3D image processing was further demonstrated in the hippocampus of a mouse model for mesiotemporal lobe epilepsy to quantify the morphological changes of astrocytes reported earlier [22], [23].

## Materials and Methods

### 1 Ethics Statement

In accordance with the policy of Grenoble Institute of Neuroscience (GIN) and the French legislation, experiments were done in compliance with the European Community Council Directive of November 24, 1986 (86/609/EEC). The research involving animals was authorized by the Direction Départementale des Services Vétérinaires de l'Isère – Ministère de l'Agriculture et de la Pêche, France and the Direction Départementale de la protection des populations - Préfecture de l'Isère-France (F. Appaix, PhD, permit number 38 09 39). All efforts were made to minimize the number of animals used and their suffering during the experimental procedure.

Animals were housed in cages with food and water *ad libitum* in a 12 h light/dark cycle at 22±1°C.

### 2 Chemicals

All chemicals were purchased from Sigma-Aldrich (France). SR101, SRB or SRG were dissolved in 0.9% NaCl (saline) at the concentration of 10 mg/ml. Fluorescein IsoThioCyanate-dextran (FITC-dextran, 70 kDa) was diluted at 100 mg/ml concentration in saline. Dyes solutions were stored at 4°C and protected from light for a maximum of one month.

### 3 Animals and surgical preparation

Rats (Sprague Dawley, postnatal day (P) 17–70, n = 32, Wistar, P17–30, n = 21, Janvier, France) and mice (adult male C57BL/6, P42–70, n = 5, Janvier, France; and FVB/N-Tg(GFAPGFP)14Mes/J, P20–45, n = 4, Jackson Laboratory, US) were used.

For *in vivo* TPLSM imaging, animals were anesthetized using isoflurane (5% for induction and 1–2% during experiments) in a 70% air, 30% O_2_ gas mixture. Their body temperature was monitored with a rectal probe and maintained at 36°C using a heating blanket. A catheter (Neoflon™, BD, USA) was inserted in the tail vein and SRB, SRG or SR101 (20 mg/kg), and/or FITC-dextran (200 mg/kg) was iv injected. A craniotomy of 2–3 mm in diameter was performed with a surgical drill above the somatosensory and motor cortex and filled with chilled artificial cerebrospinal fluid (aCSF; in mM: 126 NaCl, 2.5 KCl, 2.5 CaCl_2_, 1.2 MgCl_2_, 1.20 NaH_2_PO_4_, 25 NaHCO_3_, 1 sodium pyruvate and 22 D-glucose; bubbled with 95% O_2_ and 5% CO_2_; pH 7.4). In some case, the dura mater was carefully removed and the exposed cortex was protected by aCSF and covered with a coverslip glued on the skull. To compare our iv method with a previous report [12], 50 µl of SR101 was directly applied (5 min, 100 µM in aCSF) on the cortical surface.

For experiments on acute brain slices, animals were iv injected with SRB, SRG or SR101 as described above. One hour after injection, rats were anesthetized by ketamine injection (Imalgène®, Kétamine Virbac, France; 100 mg/kg), decapitated, and their brain was quickly removed. Coronal brain slices with a thickness of 300 µm were cut (vibratome V1000S, Leica, Germany) in ice-cold low Ca^2+^- high Mg^2+^ artificial cerebrospinal fluid (aCSF; in mM: 126 NaCl, 2.5 KCl, 0.5 CaCl_2_, 7 MgCl_2_, 1.20 NaH_2_PO_4_, 25 NaHCO_3_, 1 sodium pyruvate and 22 D-glucose; bubbled with 95% O_2_ and 5% CO_2_; pH 7.4). Following sectioning, all slices were kept at room temperature in normal aCSF (in mM: 1.20 MgCl_2_ and 2 CaCl_2_) until TPLSM imaging for a maximum of 6 hours. For comparison with the iv injection, acute brain slices from non-injected animal were incubated with SR101 (15 min, final concentration 1 µM) [24].

For the epilepsy mouse model, 6 week-old animals were injected in the dorsal intrahippocampus with 1 nmol of kainic acid (50 nl), 2 weeks before iv SRB injection, as previously described [22], [23]. The cannula was positioned in the right dorsal hippocampus (coordinates from bregma: anteroposterior [AP] = 2.0 mm, mediolateral [ML] = 1.5 mm, dorsoventral [DV] = 2.0 mm) and the kainic acid solution was injected during one minute using a micro-pump (CMA/100, Carnegie Medicine). Only animals showing signs of focal status epilepticus (e.g., mild clonies, rotations) during the 12 h following kainic acid injection were included in the study.

### 4 Two-photon microscopy and two-photon fluorescence spectra

Two-photon microscopy was performed using a LSM 7 MP (Zeiss, Germany) equipped with a 20× water-immersion objective (NA 1.0; Zeiss) and ZEN 2009 software. Laser excitation at 800–900 nm was done with a Ti:Sapphire laser system (Chameleon vision II; Coherent, UK). Fluorescence light was collected in the epifluorescence configuration. The sulforhodamines fluorescence was separated from the FITC or GFP fluorescence using a dichroic mirror (562 nm, Semrock, US). Fluorescence emissions were detected simultaneously by two non-descanned photomultiplier tubes with a 542/50 nm filter (Semrock, US) for “green” fluorescence emission and a 617/73 nm filter (Semrock, US) for “red” fluorescence emission.

Fluorescence emission spectra were measured using 100 µM solutions of SRB or SR101 dissolved in water. Fluorescence intensities of these solutions were recorded on the microscope as a function of excitation wavelengths ranging from 720 nm to 950 nm in steps of 10 nm using a constant laser power (50 mW on the sample).

### 5 Immunolabeling

Immunostaining on living brain slices was performed to characterize SRB-positive cells. Slices from SRB-injected rat brain were incubated for one hour at room temperature with either anti-NG2 antibody diluted in aCSF (1∶1000, DAKO) or anti-NeuN antibody (1∶1000, DAKO) diluted in aCSF with 0.005% Pluronic acid F-127 (Molecular Probes, Invitrogen), 0.00025% Cremophor EL (Sigma-Aldrich) and 0.05% DMSO (dimethyl sulfoxide, Sigma-Aldrich) saturated with 95% O_2_/5% CO_2_. Slices were rinsed twice in aCSF and incubated with an anti-mouse Alexa488-conjugated secondary antibody (1∶1000; Molecular Probes, Invitrogen) for one hour at room temperature. Finally, slices were put in aCSF for TPLSM imaging.

Above immunostaining method described for living brain slice could not be used for anti-S100B and anti-CD11 antibodies. Thus, a method was developed to merge astrocytes sulforhodamine staining acquired in living slice with immunostaining performed on the same slice after fixation. Rats were intravenously injected with both SRB and FITC-dextran. Blood vessels stained with fixable FITC-dextran were used as landmarks to merge images from the living and the fixed slice. Slices were then fixed with 4% paraformaldehyde in Tris Buffer Saline (TBS) with 0.3% triton-X100 (TBST) for 1 hour and incubated with 3% normal goat serum (NGS) in TBST at room temperature for 30 min. The incubation with the antibodies anti-S100B (1∶1000, DAKO) and anti-CD11 (1∶100, AbCys) in 1% NGS-TBST was performed overnight at 4°C. Then, after rinsing, slices were incubated with fluorescent secondary antibodies diluted in 1% NGS-TBST for 2 hours at room temperature: anti-rabbit Alexa488-conjugated secondary antibody (1∶1000; Molecular Probes, Invitrogen) for anti-S100B or anti-mouse Alexa488-conjugated (1∶1000; Molecular Probes, Invitrogen) for anti-CD11. Finally, slices were rinsed in TBST and mounted on slides using Vectashield medium (Vector labs, USA). Shrinkage caused by fixation was corrected using the “TurboReg” plugin [25] of ImageJ software to merge sulforhodamine staining with immunolabeling.

### 6 Electroencephalographic (EEG) recordings in freely moving rat

Sprague-Dawley rats (P25; n = 4 for SRB and n = 4 for SR101) were anesthetized with 1% isoflurane in a 70% air, 30% O_2_ gas mixture and placed in a stereotaxic apparatus. Bipolar electrodes (stainless steel wire ∅220 µm, insulated with Teflon) with a mean space of 300 µm between the 2 tips were unilaterally implanted into the S1BF somatosensory cortex (coordinates relative to bregma: anteroposterior (AP): −1.3, mediolateral (ML): −5, dorsoventral (DV): −3), into the M1 motor cortex (coordinates relative to bregma: AP: +1.6, ML: −2.5, DV: −2) and into the hippocampus (coordinates relative to bregma: AP: −3.7, ML: −2.5, DV: −3.5). These electrodes were fixed to the skull with acrylic cement and connected to a female connector. Reference electrode was made of a stainless-steel screw fixed over the cerebellum and soldered to the connector. On the following day, the behavior and EEG activity was monitored in freely moving rat pups using a video/EEG computer-based acquisition system (System Plus Evolution®, Micromed, France). The rats were first monitored for 1 h for reference. Then, either SRB or SR101 (20 mg/kg) was intravenously injected as described above and then both EEG activity and behavior were monitored during 3 days for sessions of 3 consecutive hours.

### 7 Calcium Imaging

A solution containing 5 µM Fluo-4 AM (Molecular Probes, Invitrogen, US), 0.005% Pluronic acid F-127 (Sigma-Aldrich, France), 0.00025% Cremophor EL (Sigma-Aldrich, France) and 0.05% DMSO (Sigma-Aldrich, France) was prepared in aCSF. Brain slices obtained as described above were loaded with this solution during 30 minutes at 35°C. The loading chamber was continuously bubbled with 95% O_2_/5% CO_2_. Slices were then placed in aCSF saturated with 95% O_2_/5% CO_2_ and supplemented with 1 mM sodium pyruvate, at room temperature for 30 minutes. Before imaging, slices were placed in a perfusion chamber and perfused with aCSF bubbled with 95% O_2_/5% CO_2_ at room temperature. Confocal imaging was done with an upright microscope (Eclipse E600 FN, Nikon Instruments, France) equipped with a 20× water-immersion objective (NA 0.5; Nikon) and a confocal head (confocal C1 head, Nikon). Fluo-4 was excited with an argon laser (488 nm) and emission was filtered with a 515±15 nm filter, SRB or SR101 were excited with a HeNe green laser (543 nm) and emission was filtered with a 605±75 nm filter. Images were acquired with EZ-C1 software (Nikon), in 12-bit encoded format. 512×256 images were taken at 800 ms intervals. For *in vivo* experiments, after removing the dura mater, 10 µl of a solution containing 0.9 mM Fluo-4 AM (Molecular Probes, Invitrogen, US), 2% Pluronic acid F-127 (Sigma-Aldrich, France) and 20% DMSO (Sigma-Aldrich, France) prepared in aCSF/Hepes were applied at the cortical surface for 45 min and rinsed with aCSF for 10 min. Time lapse recordings obtained were analyzed with CalSignal software [26].

### 8 Image Processing and Statistical Analysis

Image processing was performed with ImageJ software [27]. Multistacks mosaic was reconstructed using the “3D stitching” plugin [28]. Quantitative analysis of the astrocytic network was based on a 3-step procedure. First, raw images were segmented by adaptive thresholding (Bernsen) to facilitate automated cell detection. The result was then analyzed using the “Particle Analyzer” included in the BoneJ plugin [29] to determine the spatial coordinates of all detected objects. Finally, V3D software [30] was employed to visualize the raw images in 3D and to superpose markers for the detected cells in order to manually correct the result. Thus, non-detected cells can be added and artefactual objects can be removed. Normalized radial densities were calculated by dividing the histogram of the distances r between all labeled cells (bin width 3 µm) by N4πr^2^, where N is the total number of cells in the z-stacks [12]. The volume of astrocyte cell bodies was estimated using the “3D object counter” plugin [31] in ImageJ. Origin software (OriginLab) was used for statistical analyses of the epilepsy mouse model. Data are reported as mean ± s.e.m. Significant differences (*p*<0.05) between data obtained from the left (non-injected) and the right (injected) hippocampi were calculated using a Student's *t*-test.

## Results

### 1 Systemic iv injection of sulforhodamine specifically stain astrocytes in the entire brain

In a first set of experiments, we analyzed whether the systemic iv injection of SRB, SRG or SR101 specifically stained astrocytes in the whole brain of mice and rats at different times post-injection using *in vivo* TPLSM. This staining was then compared to the one obtained after local application of SR101 on the cortical surface [12].

**Figure 1:**
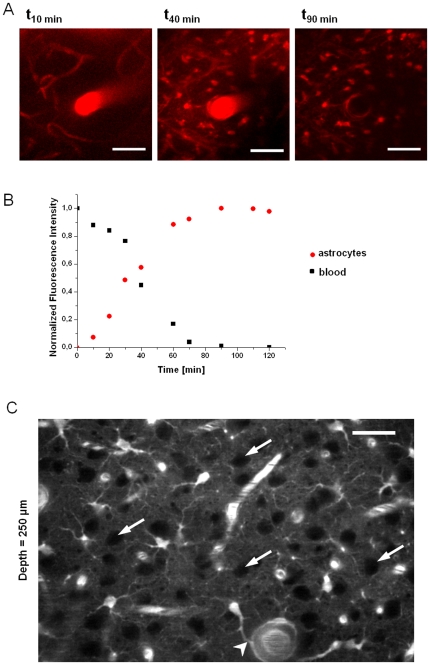
Astrocytes are progressively stained *in vivo* after an intravenous injection of sulforhodamine B. **A**) 10 min after an iv injection of SRB (P18 rat) only blood vessels were observed, 40 min after the injection both vessels and astrocytes were stained and 90 min after the injection only astrocytes could be imaged. Scale bar = 50 µm. **B**) Curve of the fluorescence intensity changes in both astrocyte and blood compartments as a function of time. **C**) *In vivo* two-photon imaging of cortical astrocytes at a 250 µm depth (P21 rat) 30 minutes after SRB (20 mg/kg) iv injection. Arrows show non-stained regions corresponding to neuron soma and the arrowhead corresponds to an astrocytic end-foot linked to a blood vessel. Scale bar = 50 µm.

Rats (18–28 days old) (n = 6) were anesthetized and injected with SRB (20 mg/kg) in the tail vein followed by a craniotomy and positioned on the motorized platform (Siskiyou, US) of the two-photon microscope. During the first 10 min, SRB was only detected in the vascular compartment (Fig. 1A). At 40 min post-injection, SRB was still found in the vasculature and accumulation occurred in numerous cells (Fig. 1A) with a shape consistent with astrocytes morphology, i.e.: cell body diameters varying between 8 and 10 µm and endfeet linked to blood vessels. 90 min after the iv injection, no SRB remained in blood vessels, whereas astrocytes cell bodies were still labeled (Fig. 1A). This pattern of staining persisted up to 5 hours after 100 µl injection of SRB (20 mg/kg). Figure 1B shows an example of fluorescence intensity changes as a function of time in both astrocytes and blood vessels. As shown in Fig. 1C, at 250 µm of depth, corresponding to the somatosensory cortex layer 2/3, SRB-stained astrocytes were observed with their endfeet linked to blood vessels (white arrowhead). Intermingled non-stained regions of larger diameter (20 µm; white arrows) were reminiscent of neurons soma suggesting that SR101 did not stain these cells after iv injection. The dose of the injected dye was chosen to obtain the best contrast and the longest staining duration. The dose of 20 mg/kg used in our experiment corresponds to the maximum amount of dye that can be injected. This quantity was limited by the sulforhodamine solubility in water and the volume that can be intravenously injected as a bolus in rats. Nevertheless, it is possible to decrease the dose but the staining may be less contrasted and may be cleared more rapidly.

Similar staining was observed following iv injection of the same dose of SR101 or SRG (n = 5; data not shown).

**Figure 2:**
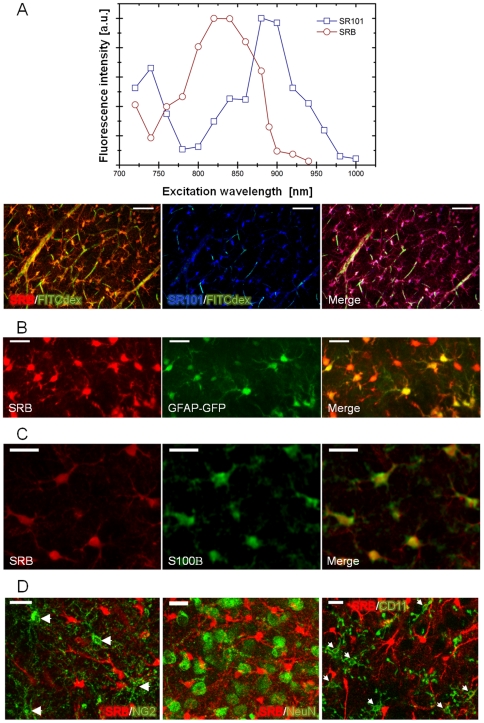
Sulforhodamine stained cells are only astrocytes. **A**) Two-photon excitation spectra of SRB and SR101 shows the possibility to excite one dye without exciting the other. Z-projection (standard-deviation) of a 100 µm stack acquired on an acute coronal brain slice of rat somatosensory cortex (P23) after iv injection of SRB/SR101 mix (1∶1) and FITC-dextran to stain vasculature. The left merged image was acquired with an 800 nm laser excitation wavelength, corresponding to SRB (orange) and FITC-dextran (green) emissions. The central merged image was acquired with a 900 nm laser excitation wavelength, corresponding to SR101 (red) and FITC-dextran (green) emissions. The right panel shows a merge of SRB, SR101 and FITC-dextran (pink corresponds to SRB and SR101 colocalization). Scale bar = 50 µm. **B**) Z-projection (standard-deviation) of two-photon microscopy images of cortical brain slices from GFAP-GFP transgenic mouse (P37) 2 h after iv injection of SRB. Left image shows SRB staining (red), central image shows GFAP-GFP expression (green) and right panel is a merge of left and central images with double-stained cells appearing in yellow. **C**) Z-projection (standard-deviation) of TPLSM images of cortical brain slices from a P18 rat. Left image: SRB astrocytes staining after iv injection (red), central image: slice immunostained with S100B antibody (green). Right panel is a merge of left and central images with double-stained cells appearing in yellow. **D**) Merge images of SRB-stained slices (red) secondarily immunostained with NG2 antibody (green, left panel), or NeuN antibody (green, central panel), or CD11b antibody (green, right panel). Arrowheads show NG2 cells or microglial cells which are not SRB-stained. Scale bar = 20 µm.

To compare the astrocytes labeling obtained using SRB or SR101, we injected intravenously both dyes in a 1∶1 mixture. The intracerebral capture of dyes was observed with TPLSM in acute brain slices using different excitation wavelengths. The maximum two-photon excitation wavelength is different for these two sulforhodamine dyes i.e.: 810 nm for SRB and between 890 to 920 nm for SR101 respectively (Fig. 2A). At 900 nm, with low laser power (less than 50 mW), emission of SRB is negligible and is close to the maximum for SR101 and vice versa at 800 nm. Therefore, two separate images could be obtained. The merge (pink in Fig. 2A) of SRB (red; 800 nm) and SR101 (blue; 900 nm) images provides evidence that these two dyes stain the same cell population. In order to confirm that the stained cells were also GFAP^+^, SRB was iv injected in transgenic mice (n = 4 animals, n = 1529 cells) expressing the green fluorescent protein (GFP) under the control of the hGFAP promoter [14]. We observed that the vast majority of GFP positive cells (89±2%) were also stained by SRB (Fig. 2B). It is important to note that the population of SRB-stained cells (red) exceeded the population of GFP-stained cells in a cortical brain slice. Indeed, we noticed that 58±2% of SRB labeled cells were not GFP positive. To characterize this second cell population, immunostainings were performed in SRB iv injected rats. Several antibodies were used, one directed against S100B (S100 calcium binding protein B), that has been shown to be specifically expressed in astrocyte cell body [32], another directed against NG2 (chondroitin sulfate proteoglycan) cells, cells able to generate oligodendrocytes and a subset of protoplasmic astrocytes [33], another directed against a neuron-specific nuclear protein (NeuN) and a later one directed against an integrin expressed on the microglial cells surface (CD11b) [34]. As shown in Fig. 2C, all SRB-stained cells also expressed S100B confirming that sulforhodamine stained astrocytes (n = 10 slices from 4 rats). On the contrary, NG2, NeuN and CD11b staining showed no overlap with SRB indicating that sulforhodamine stained cells were not NG2 cells, neurons or microglial cells (Fig. 2D).

**Figure 3:**
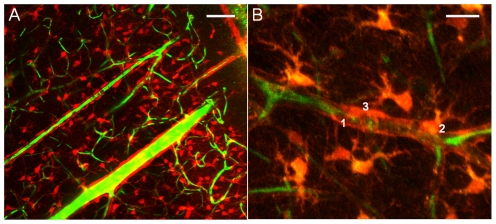
Astrocytes and blood vessels staining in acute cortical brain slices (P23 rat). **A**) Astrocytes are SRB labeled (red) whereas the vessels are stained by FITC-dextran (green). Scale bar = 50 µm. **B**) Higher magnification of image A showing that most of the surface of the vessels was covered by either astrocytic endfeet (1) or astrocytic cell bodies (2) or pericytes (3). Scale bar = 20 µm.

We further combined the SRB astrocytes labeling with a staining of the vascular network by an additional iv injection of FITC-dextran *in vivo* or 1 hour before euthanizing the rats for brain slice experiments. The labeling of the vasculature was conserved in acute brain slices (Fig. 3A) and permitted to analyze the vascular network also in subcortical areas. Depending on the vessels types, almost their entire surface was covered either by astrocyte endfeet (1) or astrocytic cell bodies (2) or pericytes (3) (Fig. 3B).

**Figure 4:**
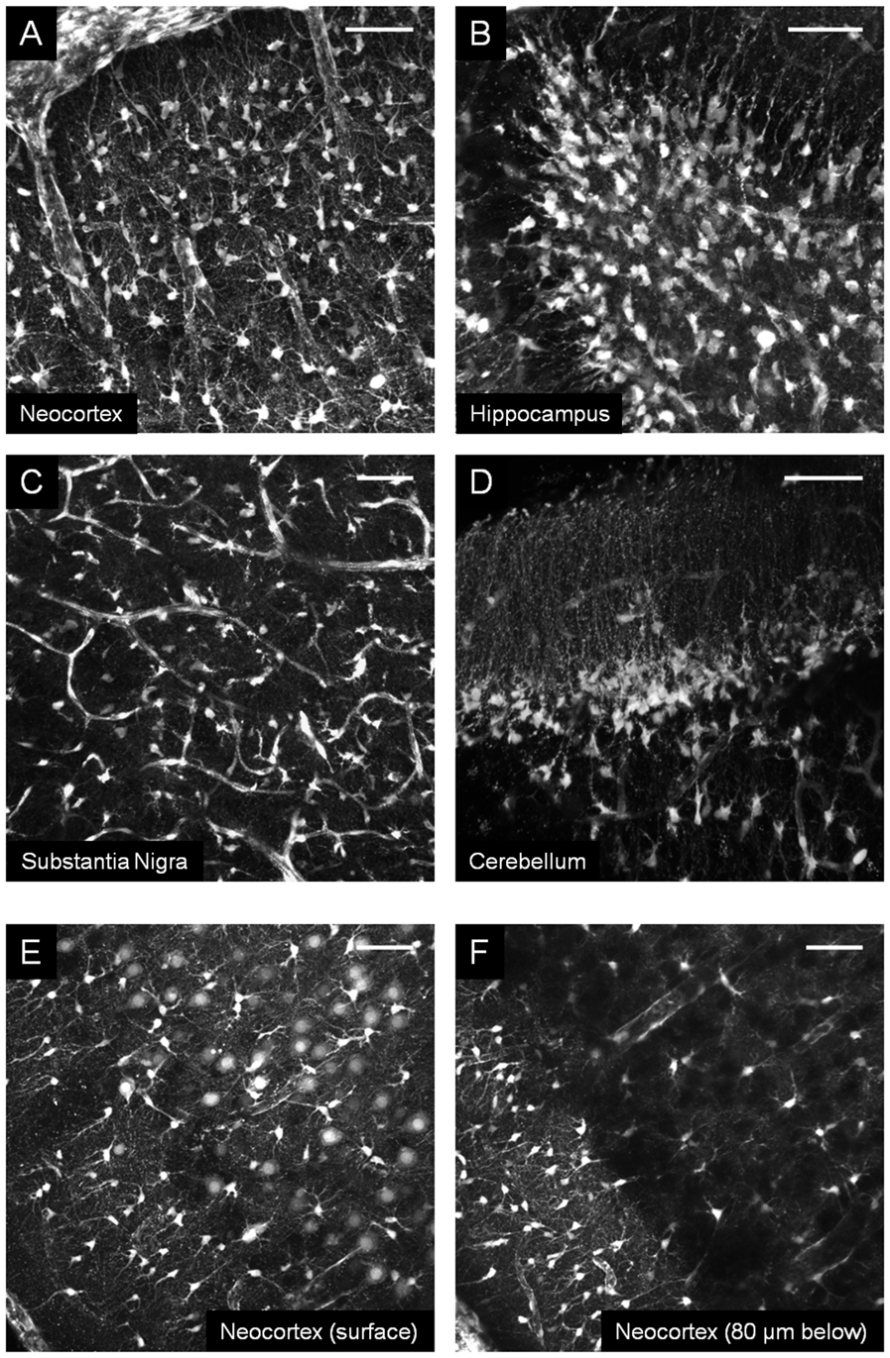
Comparison of two methods for astrocytes staining in acute brain slices (thickness of 300 µm, P18 rat). TPLSM images: **A–D**) brain slicing performed 2 h after intravenous injection of SR101 (20 mg/kg) and **E–F**) brain slices incubated 15 minutes with SR101 (final concentration 1 µM) in aCSF. Z-projections (standard-deviations) of acquired stacks: **A**) in the cortical L1 and L2/3 (stack thickness 300 µm); **B**) in the dentate gyrus (stack thickness 150 µm); **C**) in the substantia nigra pars reticulata (stack thickness 100 µm) and **D**) in the cerebellum (stack thickness 50 µm). Images collected after incubation with SR101: **E**) at the surface of the slice, and **F**) 80 µm deeper from the brain slice surface. Scale bar = 50 µm.

We hypothesized from the previous results that iv injection of SRB, SRG or SR101 stained astrocytes in the entire brain. To prove this, we prepared acute living brain slices at least 2 h after SR101 iv injection (20 mg/kg, n = 6 animals), to ensure clearance of the dye from the blood plasma. Note that *in vivo* TPLSM is limited to the maximum depth of approximately 800 µm. However, on brain slices, astrocytes could not only be detected in the neocortex (Fig. 4A), but also in the hilus of the dentate gyrus (Fig. 4B), in the substantia nigra pars reticulata (Fig. 4C) and in the cerebellum as Bergmann glia cells of cerebellar cortex (Fig. 4D). Larger images of a part of the cerebellum (Fig. S2 A), the whole hippocampus (Fig. S2 B) or all cortical layers of the S1BF (Fig. S3) show the different labeling pattern. On the contrary, when brain slices of non-injected rats (n = 4) were incubated with SR101 (15 min; final concentration 1 µM), stained astrocytes were only detected in the cortex and the hippocampus. In addition, neurons were not stained deeper than 80 µm (Fig. 4E–F).

**Figure 5:**
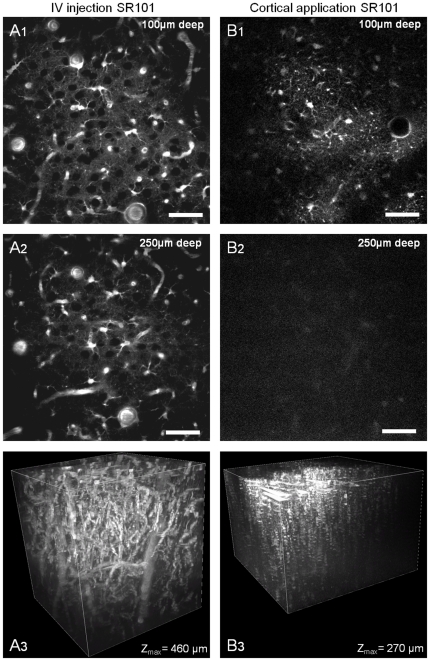
Comparison of two *in vivo* methods for astrocytes staining (P21 rat). TPLSM images acquired: **A_1–3_**) 30 minutes after SRB (20 mg/kg) intravenous injection and **B_1–3_**) 5 minutes after SR101 application (100 µM) on the cortical surface. (**A_1_**) and (**B_1_**) Images were taken at 100 µm below the pia.mater (**A_2_**) and (**B_2_**) Images were taken at 250 µm below the surface of the cortex. (**A_3_**) and (**B_3_**) show a 3D reconstruction (V3D) using the entire stack of images. Scale bar = 50 µm.

Then we compared our staining method (Fig. 5A_1–3_) with a cortical application of SR101 (Fig. 5B_1–3_, n = 3). TPLSM imaging at the surface of the cortex revealed a reduced astrocytes staining with a lower contrast in comparison to images obtained following sulforhodamine iv injection (compare Fig. 5A_1_ and B_1_). Moreover, the iv injection method showed a maximum imaging depth of 500 µm under the cortical surface in comparison to a maximum imaging depth of around 200 µm using the incubation method (compare Fig. 5A_3_ and B_3_).

### 2 Lack of adverse reactions following sulforhodamine iv injection

In the second part of this study, we verified whether the staining of the astrocytic population was compatible with physiological experiments. This staining could be helpful to discriminate astrocytes from neurons after loading with calcium indicators that accumulate in both cell types.

**Figure 6:**
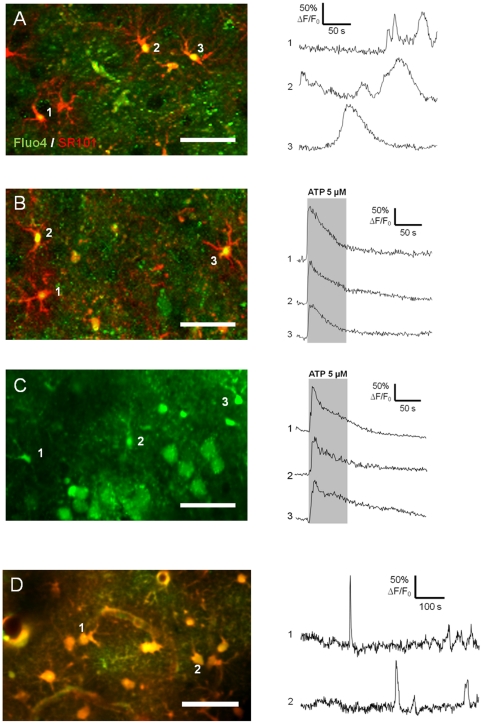
Calcium signaling in neocortical astrocytes stained with SR101. **A**) Left panel: merged confocal images of astrocytes labeled with SR101 (iv injection; red) and incubated with 5 µM Fluo-4 AM (green) in acute brain slice (P17 rat). Right panel: example of typical fluorescence variations measured in three SR101-stained astrocytes within the somatosensory cortex. **B**) Left panel: merged confocal imaging of astrocytes stained with both SR101 (red) and Fluo-4 AM (green). Right panel: example of fluorescence increase induced by ATP (5 µM) measured in three typical SR101 positive cells. **C**) Left panel: confocal imaging of astrocytes loaded with Fluo-4 AM (green). Right panel: example of fluorescence increase induced by ATP (5 µM) measured in three loaded astrocytes. **D**) Left panel: TPLSM imaging of cortical astrocytes *in vivo* 1 hour after an iv injection of SR101 (P18 rat, depth = 200 µm). Right panel: example of fluorescence variations measured in two SR101-stained astrocytes within the somatosensory cortex layer 2/3 labeled with Fluo-4 AM. Scale bar = 50 µm.

First, we have to verify that the sulforhodamine astrocyte staining did not interfere with astrocyte calcium oscillations in acute brain slices or *in vivo*. In acute brain slices, calcium oscillations in astrocytes were monitored using Fluo-4 AM, in the hippocampus, in the cortical layers 2/3 as well as in the substantia nigra pars reticulata for 150–200 seconds at 1 Hz. During a 5-min recording, more than 20% of the astrocytes recorded in the cortex showed spontaneous calcium signals with no apparent synchrony (n = 9 slices from 3 animals; 270 cells) (Fig. 6A). Spatiotemporal patterns of astrocytes spontaneous calcium signals recorded were consistent with signals obtained in other studies [35]. Moreover, application of ATP (5 µM), an agonist of P2Y receptors widely expressed on astrocytes [36], [37], induced a intracellular calcium rise in more than 40% of the SRB stained astrocytes (n = 6 slices) (Fig. 6B) similar to signals recorded without SR101 (n = 5 slices) (Fig. 6C).

Spontaneous calcium activity *in vivo* was recorded for 10 minutes using Fluo-4 AM in astrocytes of somatosensory cortex layer 2/3 at approximately 200 µm deep after SR101 astrocytes staining (Fig. 6D). We observed in our recordings that the duration and the amplitude of calcium activities were not modified by the sulforhodamine (n = 3).

Secondly, a recent study reported epileptiform activities in hippocampal slices and *in vivo* after local application of SR101 (2–5 µl, 10 µM) [38]. Others studies using intracerebral injections of smaller volumes of SR101 never reported such adverse effect [12], [35]. Therefore, we examined whether epileptic activities occurred after iv injection of SRB or SR101. Cortical and hippocampal EEG was recorded in freely moving rats (P25) for 3 h sessions during 3 days after iv injection of SRB (20 mg/kg; n = 4 rats) or SR101 (20 mg/kg; n = 4 rats). Visual analysis of the video/EEG recordings, using bipolar derivations, did not reveal any spikes, polyspikes or epileptiform discharges and no abnormal behavior reminiscent of tonic or clonic seizures (Fig. S1).

### 3 Morphological analysis of the sulforhodamine-stained astrocytic network

The ubiquitous and homogeneous astrocytes staining in the entire brain and the high contrast of images obtained following iv injection of SRB, SRG or SR101 enabled 3D morphological analysis of the astrocytic network. Similar analysis of GFAP-GFP mice was less accessible due to a heterogeneous GFAP expression in astrocytes. Images from the cortex of 5 rats (P17–19) were quantitatively analyzed to determine morphological parameters, such as (i) astrocyte volume density as a function of depth, (ii) cell body volumes and (iii) distances between neighboring cell bodies (normalized radial density).

**Figure 7:**
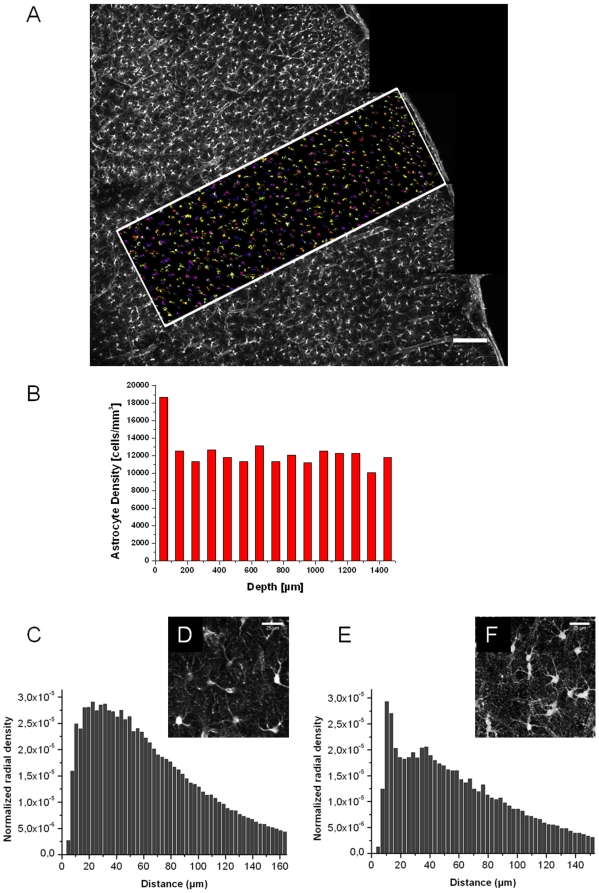
3D morphological analysis of the astrocytic network after iv injection of SRB. **A**) A multistacks mosaic acquired in the somatosensory area of a coronal acute brain slice (P17 rat). The region of interest (ROI; white rectangle) shows colored astrocytes detected using ImageJ plugins. Each color corresponds to single astrocytes cell body. Scale bar = 150 µm. **B**) Frequency histogram showing of astrocyte densities as a function of cortical depth calculated from the ROI described above. Depth was divided in 15 bins with 100 µm increments. **C**) Frequency histogram of normalized astrocytes radial densities in the cortical layer 1 and **E**) in layers 2/3. **D**) TPLSM images showing astrocytes in the cortical layer 1 and **F**) in layers 2/3. Scale bar = 25 µm.

Using ImageJ plugins for image processing, astrocyte cell bodies were first identified to determine their 3D coordinates. This enabled to calculate the number of astrocytes in a given volume as well as the normalized radial densities. A multistacks mosaic of the somatosensory cortex has been reconstructed from a coronal rat brain slice. A region of interest containing all the cortical layers has been selected for the analysis (Fig. 7A). The astrocyte volume density (n = 150–200 cells from 5 animals, P17–19) was 19.2 10^3^±0.4 10^3^ cells/mm^3^ in the cortical layer 1 (L1), and 13.7 10^3^±0.9 10^3^ cells/mm^3^ in the layers 2/3 (L2/3) (Fig. 7B). The normalized radial density histograms showed a mean cell-to-cell distance between 20 and 30 µm in the L1 (Fig. 7C). This was consistent with the uniform arrangement observed in images obtained from this area (Fig. 7D). By contrast, in the L2/3, histograms suggested the existence of two cell populations (Fig. 7E): (i) one with a normalized radial density around 10 µm and (ii) a population with a normalized radial density between 35 and 40 µm. Cells with the smallest normalized radial density appeared mostly as pairs (Fig. 7F) as previously described [12]. Further, a mean cell body volume of 960±25 µm^3^ was found in the rat neocortex (P17).

**Figure 8:**
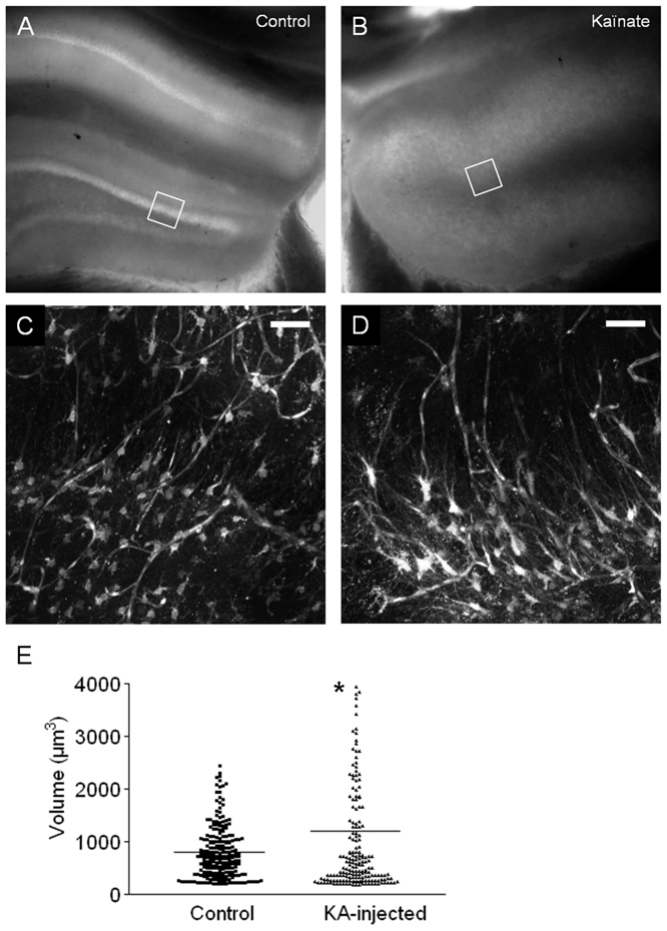
Rearrangement of the astroglial network in a mouse model of mesiotemporal lobe epilepsy. **A–B**) Bright field microscopy imaging (Nikon Multizoom AZ100, France) of acute hippocampal slices from adult mice 2 weeks after a unilateral intrahippocampal kainate injection. **A**) Contralateral non-injected hippocampus. **B**) Ipsilateral hippocampus. Note the absence of CA1/CA3 areas and the enlargement of the dentate gyrus. **C–D**) TPLSM imaging of regions indicated by white squares on (A) and (B) images, respectively, after iv injection of SR101. Scale bar = 50 µm. **E**) Column scatters representation showing the distribution of astrocytes cell body volumes at both sides of the hippocampus. The bar corresponds to the mean value.

This 3D analysis can be performed on neurological disorder models to study morphological modifications of astrocytes. We applied our method to a mouse model of mesiotemporal lobe epilepsy obtained by intrahippocampal injection of kainic acid (n = 5 mice). This model is characterized by a unilateral loss of pyramidal neurons in the CA1, CA3 and hilus regions, along with a dispersion of granule cells of the dentate gyrus and a massive hypertrophy of astrocytes with an extension of long radially-oriented processes in the ipsilateral dentate gyrus [22], [23]. Two weeks after kainic acid injection, a loss of pyramidal cells in the CA1, CA3 and hilus was observed in the ipsilateral hippocampus of all injected mice, as compared to the contralateral side (Fig. 8A–B). A drastic reorganization of the astrocytic network was observed in the ipsilateral side (Fig. 8C–D), as previously described [22]. The 3D analysis confirmed that the mean volume of the astrocytes cell body was significantly increased (1212±111 µm^3^; n = 194 cells; p<0.05) at the injected side in comparison to the contralateral side (804±33 µm^3^; n = 228 cells) (Fig. 8E). The wider distribution and the increased mean of astrocytes cell body volumes in the injected hippocampus could be attributed to a population of astrocytes that presented elongated cell bodies, see Fig. 8D and previous report [22].

## Discussion

In the present study, we propose a noninvasive method to specifically stain astrocytes in the whole rat or mouse brain *in vivo* using an intravenous injection of sulforhodamine dyes.

As observed in this study, SRB, SRG or SR101 were able to cross the blood brain barrier (BBB) after iv injection and stained cells in the brain of rat pups as well as adults and mice. Morphological characteristics of these labeled cells and immunolabeling experiments strongly suggest that sulforhodamines specifically stained astrocytes. The choice of the sulforhodamine dye (SR101 (E_m_ 606 nm), SRB (E_m_ 588 nm) and SRG (E_m_ 533 nm)) can be adapted to avoid spectral overlap with others dyes.

These SRB, SRG or SR101-astrocytes were probably stained via their endfeet close to the basal lamina of the blood vessels. The mechanism of dye trafficking from the vasculature into astrocytes remains unknown. However, considering the physicochemical properties of the sulforhodamines (i.e. a small and highly polar molecule), we hypothesize that sulforhodamine dye may diffuse passively across the blood vessel endothelium and accumulate in the perivascular space, between the astrocytic endfeet membrane and the basal lamina. Then, connexins, and especially Cx43, might enable specific uptake of sulforhodamine by astrocytes because of their localization in the endfeet membrane [13], [39]. Moreover, Cx43 is a well known hemi-channel involved in intercellular communication within the astrocytic network. Therefore the dyes might circulate over the astrocytic network via these connexins. This hypothesis is in good agreement with a previous study where carbenoxolone (a gap-junction blocker) has been shown to block the transport of SR101 between astrocytes [12].

*In vivo* staining of cortical astrocytes can also be obtained after a topical application of SR101 on the cortex [12]. Here, we showed that this local staining method of the astrocytic network was limited by the diffusion of the dye in brain parenchyma. When a SR101 intracerebral injection was performed, the dye specificity for astrocytes was improved, but the staining was spatially limited to the injection site and time-limited due to the dilution of dye in the astrocytic network. In contrast, using the iv injection method, our data suggest that all astrocytes in the entire brain were stained via the cerebral blood circulation for more than 5 hours. The sulforhodamine dye was cleared from the blood after 1 or 2 hours depending on the quantity of the dye injected, age of animals and the species. The iv staining includes not only protoplasmic astrocytes, but also marginal and perivascular ones, as well as Bergmann glia cells in the cerebellum and radial astrocytes in the hippocampus [40], [41]. Therefore, the astrocyte staining clearly appears ubiquitous and homogeneous which makes this method more suitable for whole brain astrocytes observations using e.g. *in vivo* endoscopy (not published data).

In this study, we verified whether the staining of the astrocytic population was compatible with physiological experiments. First, we showed that EEG recordings after iv injection of either SRB or SR101 did not reveal any epileptiform activity for up to 3 days post-injection. This lack of adverse effects was confirmed with the astrocytes calcium activity after iv sulforhodamine staining. This staining did not alter the astrocyte calcium oscillations *in vivo* according to the literature [11], [35].

The new iv method may enable longitudinal studies of the astrocytic network after repeated iv injections in the same animal with a chronic cranial window [42]. In these studies, iv injections are more convenient than repeated intracerebral injections that are invasive and may induce tissue lesion. This may be useful in cerebral stroke models, where changes in astrocyte morphology and function after a permanent or a transient occlusion have an impact on neuronal functions over time [43]. Another application may be the understanding of the relationships between neuronal activation and local cerebral vascular dynamics via the astrocytic network [44]. Transfected calcium indicators [45], transgenic mice with fluorescent neurons [46] and additional iv injections of FITC-dextran and sulforhodamine for the staining of respectively the cerebral vasculature and astrocytes might be used to avoid intracerebral injections. The iv method leads to a more exhaustive labeling of astrocytes in comparison to transgenic animals. Indeed, SRB iv injection in GFAP-GFP transgenic mice revealed that only 40% of the SRB stained astrocytes were GFP^+^. These data confirm the fact that only a subpopulation of astrocytes can be detected in transgenic mice, likely due to variation in the promoter expression and/or GFP protein levels in the cells [47]. At the opposite, 10% of GFAP-GFP cells were not stained by SRB in transgenic mice. These cells could be attributed to astrocytic precursors such as NG2 cells that do not possess functional endfeet [48], [49] for SRB uptake from the cerebral vasculature.

The current method can also be used for experiments on acute brain slices of mice and rats. The commonly used method consists in the incubation of brain slices with sulforhodamine [24], leading to a neuronal staining at the surface of the slice. This uptake of the sulforhodamine by neurons *in vitro* might be due to the damage or swelling of neurons during the slice preparation [50]. On the contrary, when acute brain slices were prepared after our iv staining method, astrocytes were observed in the whole slice from any brain regions without any neuronal staining.

Comparing to others, our method results in a homogeneous astrocytes staining in the entire brain and its high contrast allowed us to perform a 3D quantitative analysis of astrocytic network. In this study, the astrocytic network in a mouse model of epilepsy was quantitatively analyzed, confirmed and extended previous immunohistochemical observations in the same model [22].

In conclusion, astrocytes staining using the iv injection method is specific, ubiquitous, exhaustive and appears a powerful tool that can be combined with other methods to study the astrocytes functions in different rodent species. In particular, our method opens new perspectives for calcium imaging and patch-clamp techniques on astrocytes *in vivo* or *in vitro*. It will also help in pathophysiological studies of astrocyte dysfunction in neurological disorders such as stroke, epilepsy, Alzheimer and Parkinson's diseases [51].

## Supporting Information

Figure S1 **EEG recordings of freely moving rats after iv injection of SRB or SR101.** EEG in both somatosensory and motor cortex and in hippocampus (rat P25) after an intravenous injection of SRB did not reveal any epileptic seizures even after recording sessions of 3 h during 3 days.
(TIF)

Figure S2 **Multistacks mosaics of large brain areas showing different pattern of sulforhodamines labeling.** **A**) Large view of the cerebellum (3224×2212 µm) showing astrocytes and blood vessels stained after iv injection of both SRB (red) and FITC-dextran (green). **B**) Astrocytes staining in the whole hippocampus (2982×1882 µm) after iv injection of SR101.
(TIF)

Figure S3 **Multistacks mosaic of astrocytes staining in all cortical layers of the S1BF cortex after iv injection of SR101 (2229×2194 µm).**
(TIF)
