# Supplementary figures and images for: Specific In Vivo Staining of Astrocytes in the Whole Brain after Intravenous Injection of Sulforhodamine Dyes
Source: PLoS One. 2012 Apr 11;7(4):e35169. doi: 10.1371/journal.pone.0035169 (PMC3324425; doi:10.1371/journal.pone.0035169)

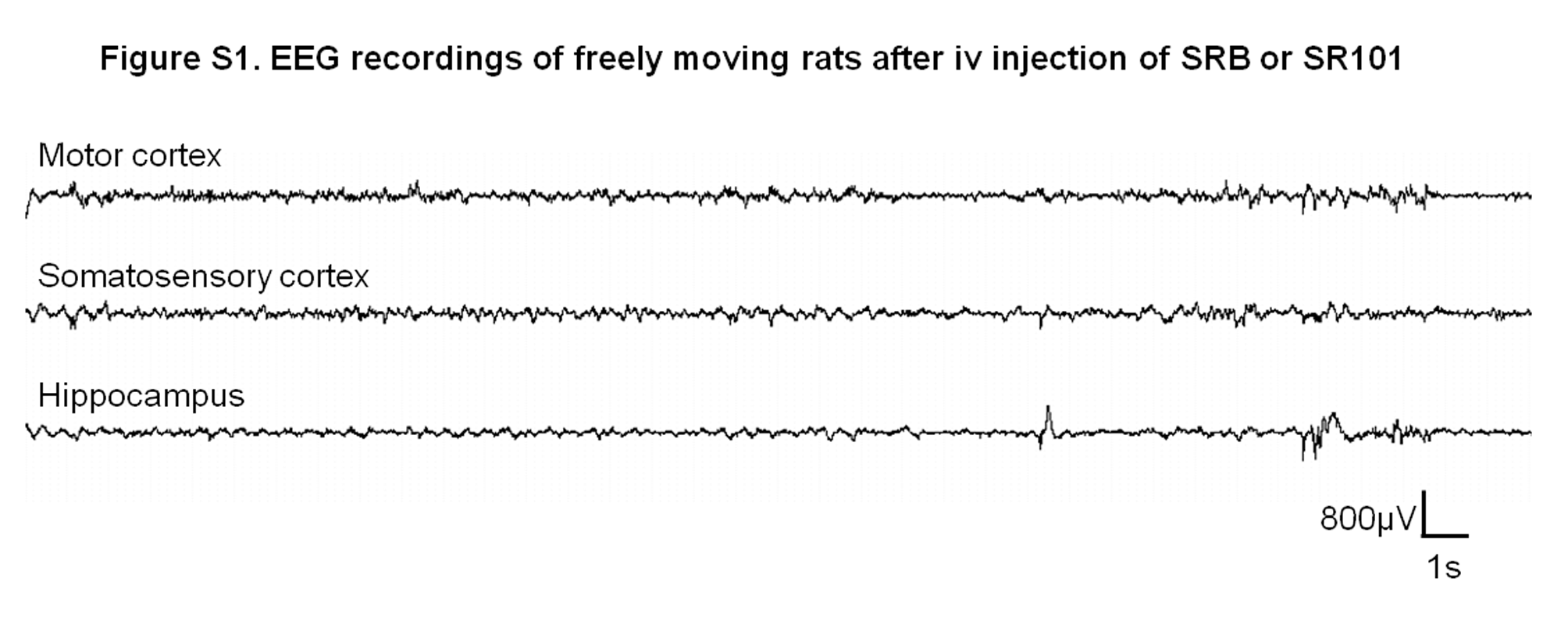

Supplement: Figure S1 — EEG recordings of freely moving rats after iv injection of SRB or SR101. EEG in both somatosensory and motor cortex and in hippocampus (rat P25) after an intravenous injection of SRB did not reveal any epileptic seizures even after recording sessions of 3 h during 3 days. (TIF) [file pone.0035169.s001.tif]

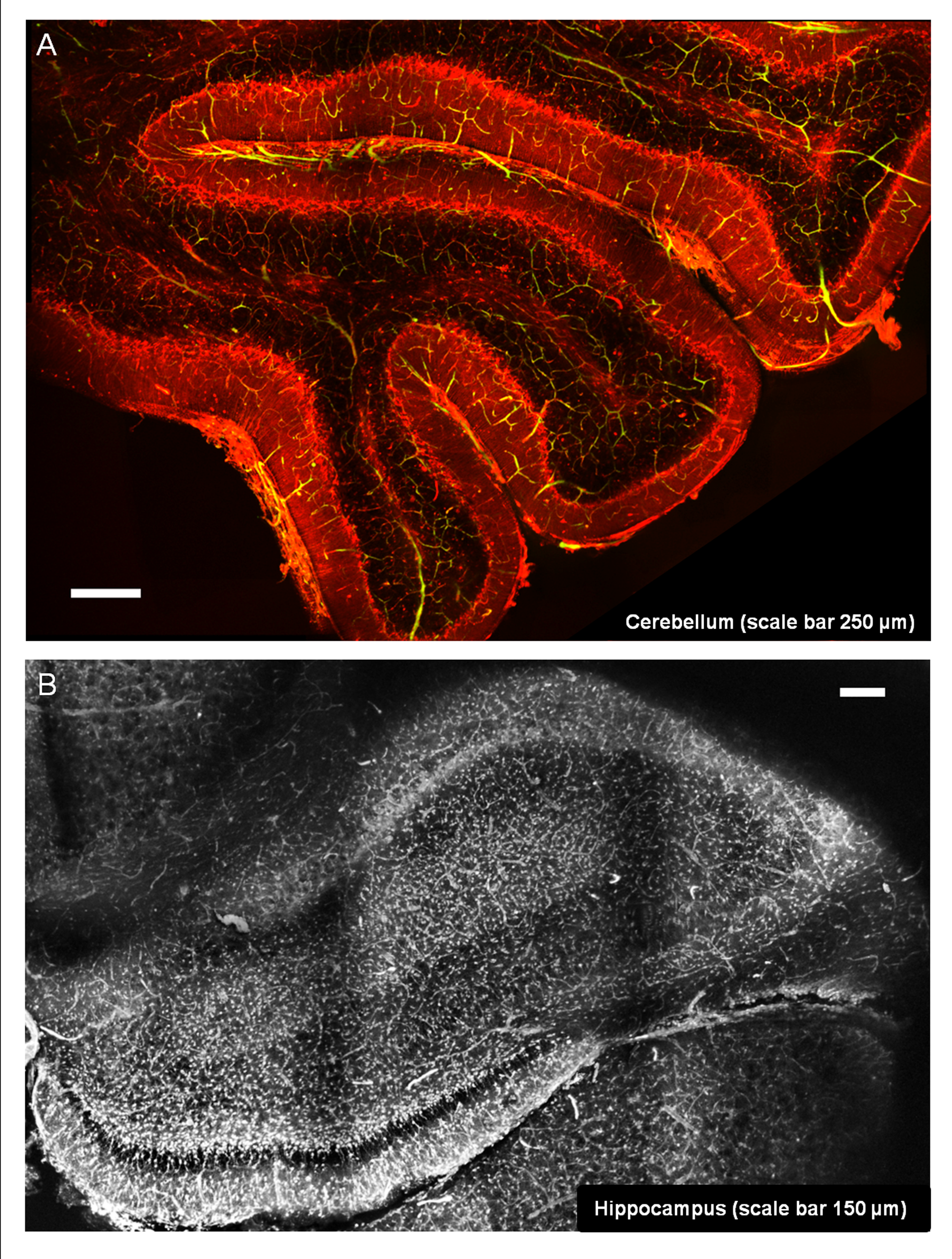

Supplement: Figure S2 — Multistacks mosaics of large brain areas showing different pattern of sulforhodamines labeling. A) Large view of the cerebellum (3224×2212 µm) showing astrocytes and blood vessels stained after iv injection of both SRB (red) and FITC-dextran (green). B) Astrocytes staining in the whole hippocampus (2982×1882 µm) after iv injection of SR101. (TIF) [file pone.0035169.s002.tif]

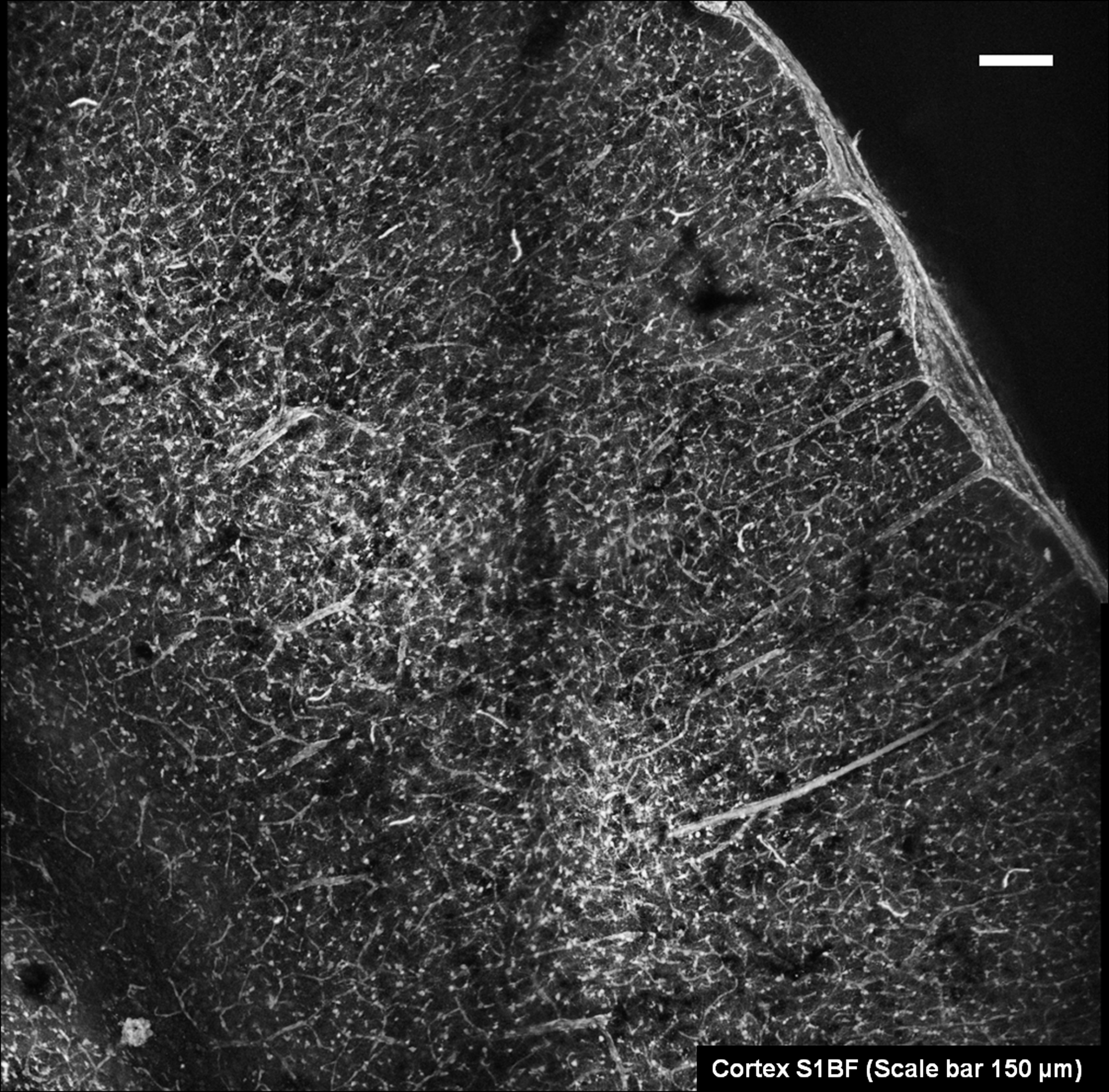

Supplement: Figure S3 — Multistacks mosaic of astrocytes staining in all cortical layers of the S1BF cortex after iv injection of SR101 (2229×2194 µm). (TIF) [file pone.0035169.s003.tif]
